# Twist-assisted high sensitivity chiral fiber sensor for Cd^2+^ concentration detection

**DOI:** 10.1016/j.isci.2022.105245

**Published:** 2022-09-29

**Authors:** Boyao Li, Yaoyao Liang, Aoyan Zhang, Lu Peng, Jinghua Sun, Guiyao Zhou

**Affiliations:** 1 School of Electronic Engineering and Intelligentization, Dongguan University of Technology, Dongguan, Guangdong, 523808, China; 2 Guangzhou Key Laboratory for Special Fiber Photonic Devices and Applications, School of Information Optoelectronics Science and Technology, South China Normal University, Guangzhou, Guangdong 510006, China; 3 Centre de Nanosciences et de Nanotechnologies, CNRS, University Paris-Sud, Université Paris-Saclay, C2N – 10 Boulevard Thomas Gobert, Palaiseau 91120, France; 4 School of Physical Sciences, The University of Adelaide, Adelaide, SA 5005, Australia

**Keywords:** Fiber optics, Sensor system, Sensory evaluation of food products

## Abstract

The ability to accurately and cost-friendly monitor heavy metals in environmental solutions such as drinking or tap water is of great significance to the human health. We report a twisted fiber-based sensing mechanism that can realize highly accurate detection of Cd^2+^ concentration in water solution. The basic design is a twisted single-core fiber simply coated with a propylene thiourea membrane that can absorb Cd^2+^. Due to the twisting effect, light in the core can scatter into the cladding, yielding optical coupling and interference. We experimentally prove that both positions and amplitudes of interference dips in the sample transmission spectrum can effectively and linearly response to the change of Cd^2+^ concentration at the level of 10^−11^ mol/L. With bimodal calibration, such sensor can realize accurate and real-time monitor in a stable and nontoxic way. These excellent characteristics indicate promising potential in the field of biochemical and integrated optical sensing.

## Highlights

• A twisted fiber-based high-sensitivity sensing mechanism
• Easy preparation, low-cost, and environmental-friendly
• Real-time stable monitor of Cd^2+^ concentration

## Graphical abstract

**Figure:**
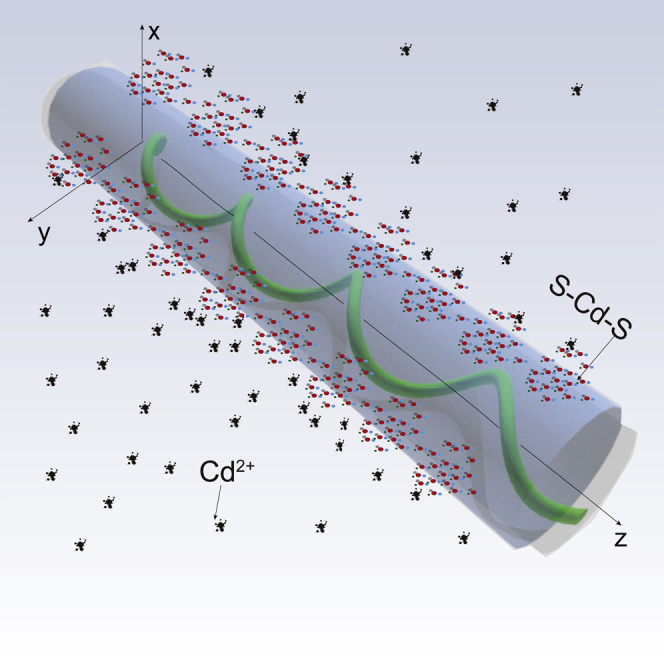

## Introduction

The detection of Cd^2+^ is indispensable in many fields especially in industry (Tiwari et al., 2017) and agriculture (Khan et al., 2017), as the Cd^2+^ contaminants remaining in water or soil could be absorbed by human through food chain (Borah et al., 2018). It is worth noting that Cd^2+^ metabolizes very slowly in the human body and is difficult to be eliminated by excretion (Järup and Akesson, 2009). When the Cd^2+^ accumulation in the human body reaches a certain concentration, it will induce a series of serious diseases such as cancer (Jiang et al., 2008; Waisberg et al., 2003). Therefore, an accurate measurement of Cd^2+^ has great significance in healthcare (Lelet et al., 2017; Pal and Pal, 2017).

To this end, the method of measuring Cd^2+^ includes inductively coupled plasma-atomic emission spectrometry (Zhang and Hu, 2012), solid phase extraction (Manzoori et al., 2007; Zhou et al., 2017), room temperature ionic liquids measurement (Aguilera-Herrador et al., 2008), atomic absorption spectroscopy (Pourreza and Ghanemi, 2010; Amiri-Yazani et al., 2019; Nazerdeylami and Zare-Dorabei, 2019; Zare-Dorabei et al., 2016), fluorescent probe method (Dai et al., 2017), and so on. Although these methods can achieve high-accuracy and effective detection of Cd^2+^ concentration in solution, they are highly dependent on expensive instrumentation, time-consuming in measurement, and not portable for *in situ* analysis, especially for real-time monitoring, which limit their applications and hinder integration into compact and cost-effective systems. What’s worse, measurement reagents of these methods are usually toxic and may cause secondary pollution. Therefore, how to establish a low-cost and efficient way to measure Cd^2+^ in solution is in highly demand.

In parallel, optical fiber sensors have attracted widespread attention due to their advantages of compact size, high stability, repeatability, and electromagnetic immunity in remote sensing (Zheng et al., 2018; Ding et al., 2019; Li et al., 2018a, 2018b, 2019a, 2019b; Cai et al., 2020; Loyez et al., 2022; Yesudasu et al., 2021; Polley et al., 2019). Combined with biochemical sensing, fiber can provide cheap and remote online measurement of chemicals or biochemicals. So far, various optical fiber-based sensing techniques have been demonstrated based on mechanisms ranging from surface plasmonic resonance (Mai et al., 2019; Zeni et al., 2020; Guo et al., 2017; Zhou et al., 2022), localized surface plasmonic resonance (Dhara et al., 2019), to lossy mode resonance (Zubiate et al., 2019; Chiavaioli et al., 2018). However, due to the extra need of litho-writing and depositing processes for metallic layers on fiber surface at high temperature, the plasmon-based techniques are usually complex for samples to fabricate. Besides, up to now, to the best of our knowledge, few work on the high-accuracy detection of Cd^2+^ was reported based on the twisted fiber.

**Figure 1:**
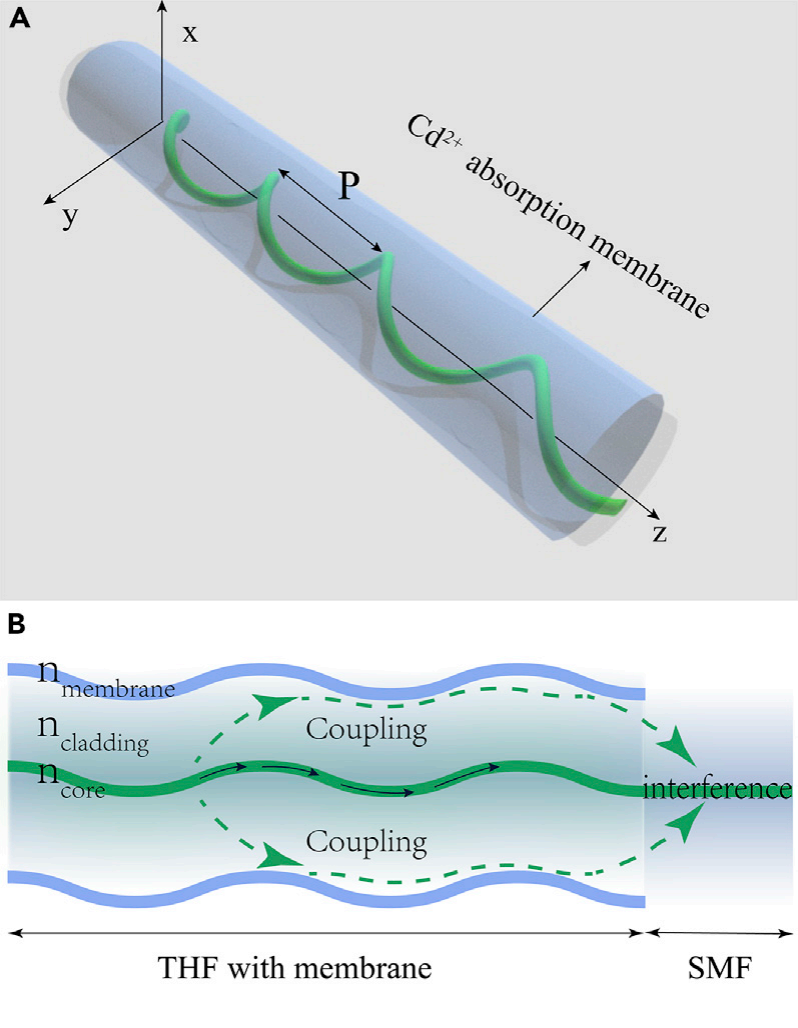
Schematic diagram of THF-based sensor structure (A) THF coated with specific membrane sensitive to Cd^2+^. (B) The sensing mechanism of the proposed sensor.

Here, we demonstrate a twisted fiber-based sensing mechanism that can realize highly sensitive *in vitro* detection of Cd^2+^ concentration in water solution. The structure is shown in Figure 1A, a twisted helical fiber (THF) simply coated with propylene thiourea membrane that can absorb Cd^2+^. Due to the helical property, light in the core can leak into the cladding; the simultaneous existence of two optical pathways, either directly through the core or via cladding region back to the core, will make coupling and give rise to the interference in the single-mode fiber (SMF). We show experimentally that the interference dips in the transmission spectrum of the sample can effectively and linearly response to the change of Cd^2+^ concentration at the level of 10^−11^ mol/L, both for dip positions and amplitudes. Through bimodal calibration, such sensor can realize accurate and real-time monitor in a stable and nontoxic way. We envision such easy preparation, low-cost while high-accuracy and environmental-friendly sensing mechanism is promising in integrated optical sensing measurement fields.

## Results

### Theoretical analysis

In contrast to conventional optical fiber where light is well confined in core region and cladding mode can be ignored, light in the core of twisted fiber would leak into the cladding region due to the twist effect, in this case the cladding mode become nontrivial. As shown in Figure 1B, there will exist two optical paths for the two modes to propagate in the twisted fiber, one is core mode path where light directly transmits through the core (the black arrows), the other is cladding mode path (dashed green arrows) where light leaking from core propagates in the cladding. During the propagation, the two modes will couple to each other in THF. We assume the $E_{1}$ is the amplitude of the core mode, the effective amplitude of the membrane-coated cladding mode is represented by $E_{2}$. Then, the coupling phenomenon between $E_{1}$ and $E_{2}$ can be described as follows:

(Equation 1) $$\begin{array}{l} \frac{dE_{1}}{dz}=i\beta_{1}+\kappa E_{2}\\ \frac{dE_{2}}{dz}=i\beta_{2}+\kappa E_{1} \end{array}$$

In above equation, $\beta_{1}=\frac{2\pi }{\lambda }n_{ceff}$ and $\beta_{2}=\frac{2\pi }{\lambda }n_{caeff}$ are propagation constants corresponding $E_{1}$ and $E_{2}$, respectively. $n_{caeff}$ and $n_{ceff}$ are the effective refractive indexes that depend on the refractive indexes of core ($n_{core}$), membrane ($n_{membrane}$), and cladding ($n_{cladding}$). The relation between them is as follows:

(Equation 2) $$\begin{array}{l} n_{caeff}=q_{1}n_{membrane}+q_{2}n_{cladding}\\ n_{ceff}=q_{3}n_{core} \end{array}$$

In which, $q_{1}$ $q_{2}$, and $q_{3}$ represent the weight factors for the effective indexes. Note that, the $n_{core}$ is constant when the thickness and refractive index of membrane change. It means the changes of membrane will not cause change in the coupling wavelength but in coupling loss. Owing to that the refractive index of membrane will change with the Cd^2+^ concentration of the surrounding solution, at special case when the propagation constants of core and membrane-coated cladding are equal ($\beta_{1}=\beta_{2}$), light from two pathways ($E_{1}$ and $E_{2}$) will maximumly couple. By connecting THF with an untwisted single-mode fiber (SMF), the effective cladding mode and core mode of THF will propagate forward and generate interference in the core region of SMF, in which the total intensity can be described:

(Equation 3) $$I={\left(E_{1}\;\cos\left(\varphi_{0}\right)+E_{2}\;\cos\left(\varphi_{0}+\Delta \varphi \right)\right)}^{2}$$

where $\varphi_{0}$ the initial phase of the core light, and Δφ the phase difference between core and cladding, defined as:

(Equation 4) $$\Delta \varphi =\frac{2\pi }{\lambda }\left(n_{ceff}-n_{caeff}\right)L$$

in which L is the interference length and is the incident wavelength. It is worth noting that the $n_{ceff}$ is related to the refractive index and twisted period of the core (Li et al., 2019a, 2019b). $n_{caeff}$ is not only related to the refractive index and twisted period of the cladding itself but also to the refractive index of membrane. The specific surface area of the inner side of the membrane will increase at the presence of the deformation of the membrane attached to the twisted cladding surface. When the refractive index of the corresponding membrane changes with different Cd^2+^ concentrations, the variations of the effective cladding refractive index ($n_{caeff}$) and optical path of cladding will be amplified due to the torsional deformation inside the film, leading to larger variation of interference intensity. Therefore, the twisted fiber-based sensing mechanism can be reasonably established on the response of interference spectrum, such as the sensitivity of interference dip positions or the amplitudes, to the Cd^2+^ concentration in the sample solution.

### Sample fabrication

**Figure 2:**
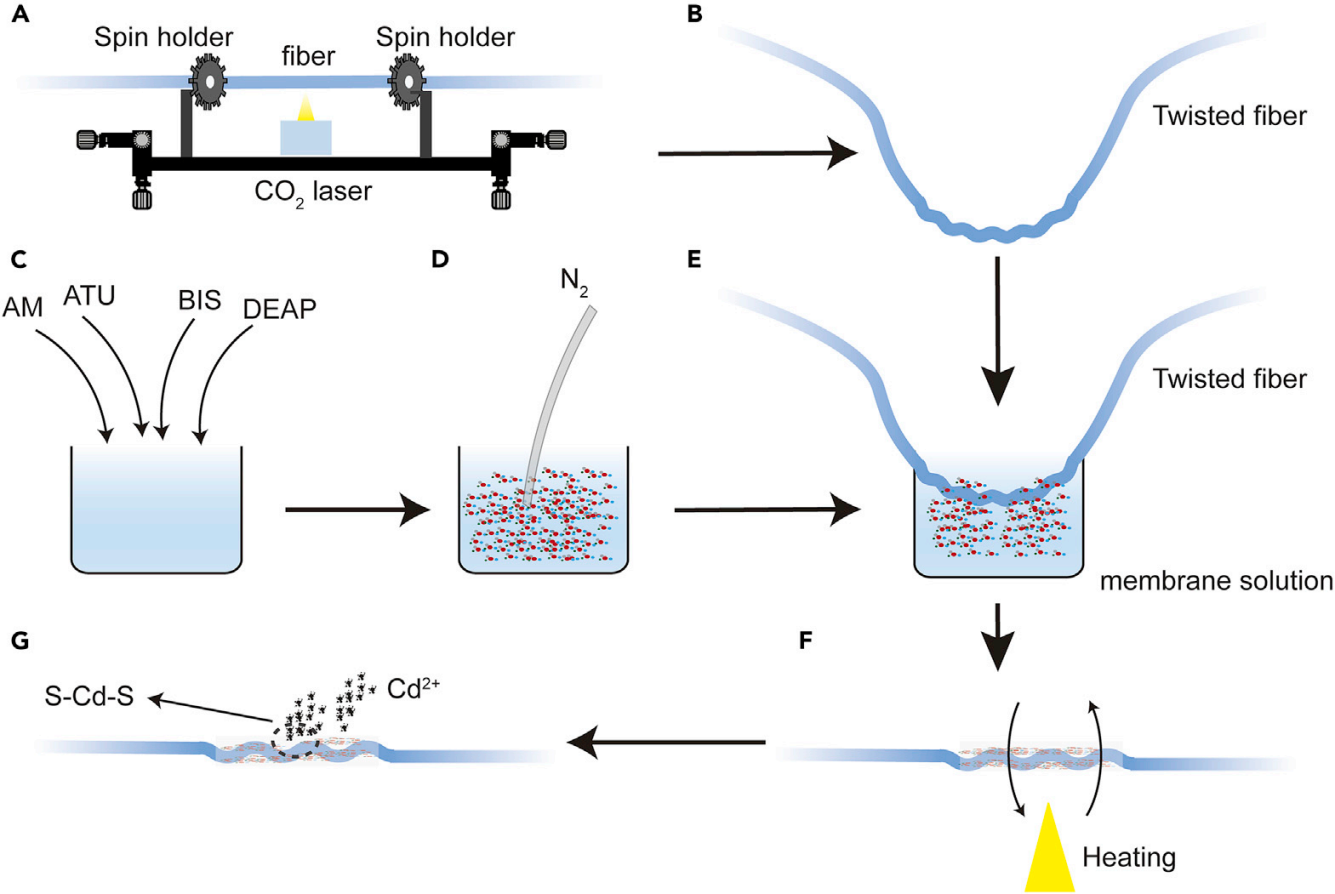
Sketch of sample preparation (A) Single-mode fiber twisting setup. (B) Twisted fiber. (C) Membrane component, (D) N_2_ Pumping. (E) Immerse the twisted optical fiber sample in the membrane solution. (F) Rotate and heat the membrane region of the twisted fiber. (G) Cross-linked "-S-Cd-S-" structure is formed due to the absorption of Cd^2+^.

THF was fabricated by twisting single-mode fiber utilizing LZM 100 splicer. Twist period of THF is set to 500 μm, and twisted length is 5 *mm*. Figure 2 shows the schematic of THF with a propylene thiourea membrane (Joseph et al., 2007) which can effectively absorb Cd^2+^ in an acidic environment. The sensing membrane is composed of acrylamide (AM, 99%), 1-allyl-2-thiourea (ATU), N, N'-methylene bisacrylamide (BIS), and 2, 2-diethoxyacetophenone (DEAP, ≥ 95%). The mixed solution was prepared by mixing AM (32.4 *mmol*), ATU (15 *mmol*), BIS (2.5 *mmol*), and DEAP (10 *μL*) in 1 *mL* H_2_O for 3 h. N_2_ is continuously pumped into the mixed solution for 10 min to remove oxygen and obtain a uniform solution. When the solution is ready, immerse the twisted optical fiber sample in the solution and take it out slowly. Then rotate the fiber slowly and heat it. Finally, a uniform and transparent membrane is obtained. The thiourea group can efficiently combine with Cd^2+^ in the polymerization system due to its high electron cloud density (Luliński et al., 2014). On the propylene thiourea membrane, a new cross-linked "-S-Cd-S-" structure is formed due to the absorption of Cd^2+^, which changes the refractive index of the membrane.

### Experimental results

**Figure 3:**
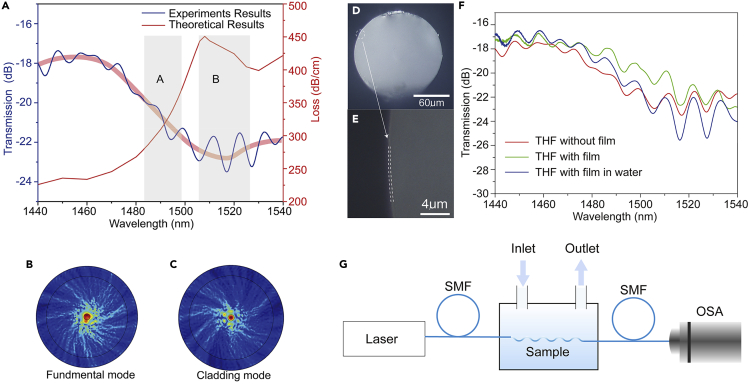
Analysis of transmission coupling characteristics of twisted fiber (A) Comparison of experimental transmission spectrum and theoretical loss spectrum of twisted fiber. Region “A” is the fast-changing region of the coupling between fundamental mode in core and cladding mode, region “B” is the maximum coupling region, and the corresponding light red curve is the envelope of the experimental transmission spectrum. It can be seen that the transmission spectrum is affected by the coupling modulation of core mode and cladding mode. (B) Fundamental and (C) cladding mode fields at the region B in twisted fiber core calculated by numerical simulation, respectively. (D) The cross section of THF coated with a film, with a magnified image in (E). (F) Comparison of transmission spectrum of the twisted fiber before (red) and after (green) coating, and the transmission spectrum of the coated sample immersed in water (blue). (G) The sketch of experimental setup used to measure the Cd^2+^ concentration in sample solution.

In order to verify the above sensing mechanism, we first obtain the theoretical loss spectrum and experimental transmission spectrum of THF with above twisted period (500 μm) before the membrane coating. It can be found in Figure 3A that the theoretical loss spectrum of fundamental mode of twisted fiber is in good agreement with measured transmission spectrum (areas with high loss correspond to low transmittance). Besides, the experimental transmission spectrum fluctuates up and down around the envelope of the light red curve, which proves the existence of coupling between the core mode and the cladding mode. It should be noted that the quality factors of interference dips in region “A” and “B” are different in the experimental spectrum of Figure 3A, region “A” is the fast-changing coupling region where the quality factors are much lower than that in region “B” (maximum coupling region). Figures 3B and 3C are the numerical calculated field distributions of core mode and cladding mode in region “B”, respectively. The similar field distribution further proves the coupling between the two modes. These phenomena indicate that the twisted fiber-based sensing mechanism can be established on the interference modulation of the coupled core mode and cladding mode.

After verifying the mode interference effect in THF samples, we further coat a Cd^2+^ absorption membrane on the surface of THF. The cross-section of membrane-coated THF is shown in Figure 3D, a magnified image displayed in Figure 3E. The thickness of related sensing membrane is 400 nm. The comparison results of transmission spectrum of THF before and after coating are recorded as shown in Figure 3F. It can be seen multiple resonant dips appear due to the interference between cladding mode and core mode. It is worthy to note that the loss of THF with membrane is lower than that without membrane, which is because that light initially leaked into the air is localized in the membrane and further transmitted to the inner part of the optical fiber. When the surrounding solution switching from air to water, due to smaller refractive index contrast between the membrane and water, light originally in the membrane will further leak into the water, leading to more losses as shown in Figure 3F. Since the S-Cd-S bond is only designed for Cd^2+^, impurities in aqueous solutions will hardly influence the interference property of the transmission spectrum. To verify it, we introduced calcium ions of different concentrations, that will deposit on the fiber surface, in the sample solutions. As shown in Figure S1 (see supplementary material), the transmission spectrum is almost consistent with that in water, which proves the good target detection of Cd^2+^ for proposed S-Cd-S bond structure and robust immune property to impurities that lead to low refractive index changes in sample solution.

**Figure 4:**
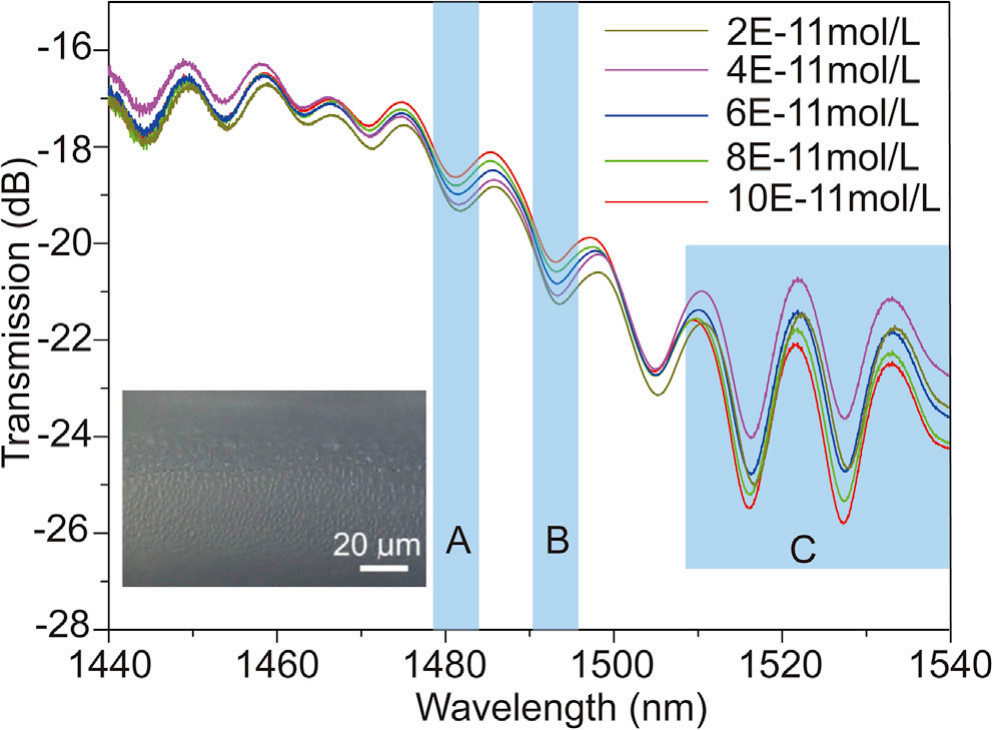
The change of transmission spectra with various Cd^2+^ concentration The refractive index of all solutions is 1.333035.

For realizing the inline detection, the sensing performance was tested by the setup as sketched in Figure 3G. The light of customized broadband light source goes through SMF to THF; the final signal was recorded by optical spectrum analyzer. Besides, we used the syringe inject analyte into a liquid chamber and pumped analyte out utilizing syringe as well. The prepared fiber sensor responding to different concentrations of Cd^2+^ solution is shown in Figure 4, in which we can clearly see the transmission spectrum changes with the concentration of Cd^2+^. Note of, the tests for different concentrations are conducted at temperature of 26°C and the relative air humidity of the corresponding surroundings is 38. The inset of Figure 4 is a side view of the sample immersed in Cd^2+^ solution (10^−11^ mol/L), which proves the interaction between Cd^2+^ and the membrane, leading to a rough surface. According to analysis in Figure 3A, “A” and “B” zones in Figure 4 are the rapidly changing areas, where the coupling of core mode and cladding mode is in a balance, which can accurately reflect the sensitivity of dip positions or intensities to the outside environment in a regular way. On the contrary, we can find that the interference dip positions in region “C” almost stay the same at different Cd^2+^ concentrations; although the intensity change of interference dips is significant, the way it changes is not as easy as in region “A” and “B” to trace. Therefore, considering the linearity and practical application of the sensor, “A” and “B” regions in Figure 4 are the main analysis parts in this research. It can be seen from the figure that with the increase of the concentration, the corresponding characteristic dips of the transmission spectrum in “A” and “B” have blue shifted, and related transmission of dips is increased. On the one hand, the increase of the concentration leads to the increase of the refractive index of the membrane, and the corresponding phase matching point can be reached earlier in short wavelength compared to that in long wavelength, so the blue shift occurs. The increase of the concentration leads to the increase of the roughness of the membrane surface, which also enhances the scattering of light at the interface, in this case more outgoing light at the interface returns to the core due to scattering, the corresponding dip transmission increase.

**Figure 5:**
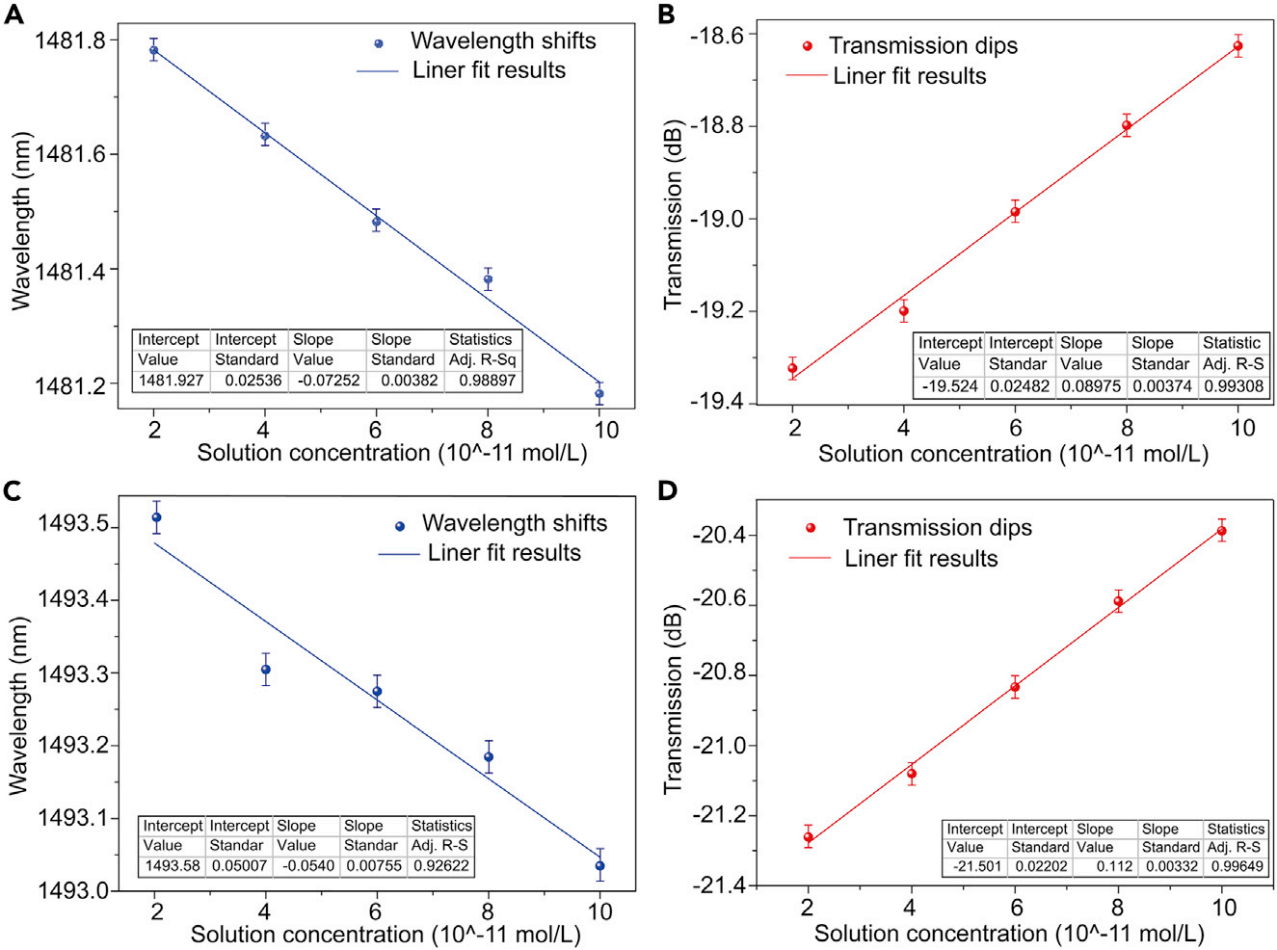
Sensitivity characterizations of proposed sensor (A), (C) Wavelength sensitivities and (B), (D) amplitude sensitivities in “A” and “B” zones of Figure 4, respectively. The times used in response and recovery are 10 and 50 s, respectively.

Then, five measurements for each experiment under the same environment according to the concentration gradient in Figure 4 were conducted. In addition, the sensitivity and error bar were calculated. Firstly, wavelength sensitivity and amplitude sensitivity in “A” zone are analyzed. Figure 5A is the wavelength of the sample transmission spectrum shifts with the increasing of Cd^2+^ concentration. It can be observed that there is a good linear trade-off between the concentration and the wavelength, and the correction coefficient of determination is 0.98897, the corresponding sensitivity can reach to 0.07252 nm/($10^{-11}mol/L$). When the resolution of the spectrometer is 0.01 nm, the sensor can distinguish the Cd^2+^ concentration at the order of magnitude of $10^{-12}mol/L$. In addition, the amplitude sensitivity is also analyzed as shown in Figure 5B. The correction coefficient of determination is 0.99308, and corresponding sensitivity can reach to 0.08975 dB/($10^{-11}mol/L$). Therefore, wavelength and amplitude of dual modulation sensor is achieved.

Similarly, as shown in Figures 5A and 5B, the wavelength sensitivity and amplitude sensitivity of region “B” in Figure 4 can reach 0.0540 nm/($10^{-11}mol/L$) and 0.112 dB/($10^{-11}mol/L$), respectively. In order to make the calibration of concentration change more accurate, the concentration variation can be connected with the position and amplitude changes of resonant dips in two regions at the same time. The demodulation function of concentration change is as follows

(Equation 5) $$\Delta c=\frac{1}{2}\left[\begin{array}{ll} \Delta W_{a} & \Delta I_{a} \end{array}\right]\left[\begin{array}{l} 1/0.07252\\ 1/0.08975 \end{array}\right]+\frac{1}{2}\left[\begin{array}{ll} \Delta W_{b} & \Delta I_{b} \end{array}\right]\left[\begin{array}{l} 1/0.054\\ 1/0.112 \end{array}\right]$$

where $\Delta W_{a}$ and $\Delta I_{a}$ represent changes of wavelength and amplitude in zone “A”; $\Delta W_{b}$ and $\Delta I_{b}$ represent relevant changes in zone “B” (in Figure 3A). Δc is variation of Cd^2+^ concentration, and the unit is $10^{-11}mol/L$.

## Discussion

### Stabilization test

**Figure 6:**
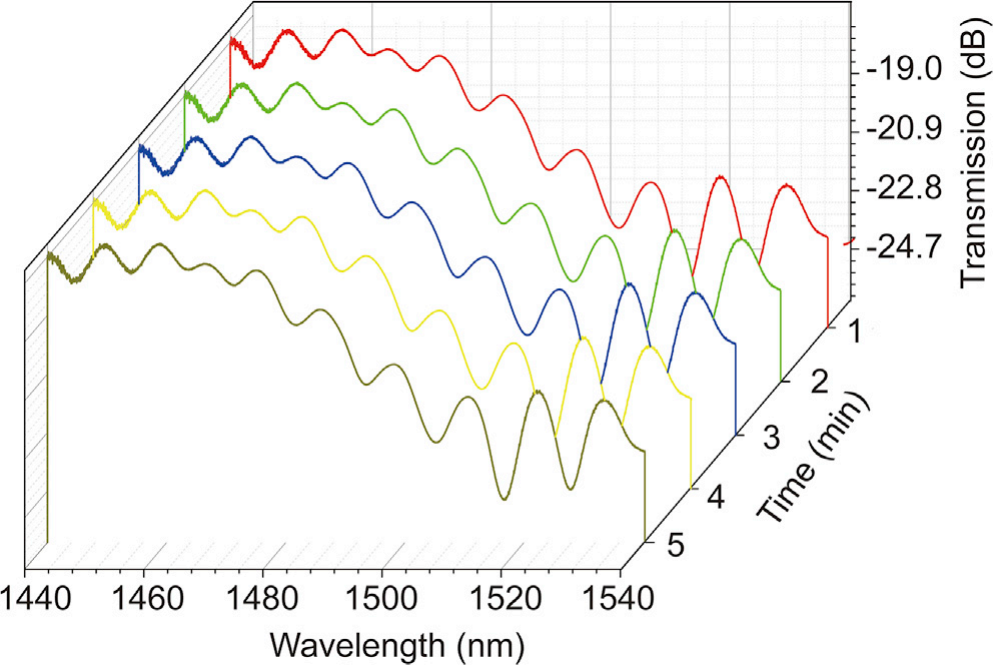
Stability test The time stability of the sensor was tested when the concentration of Cd^2+^ was $2\times 10^{-11}mol/L$.

In order to further explore the performance of the prepared fiber sensor, the time stability of the sensor is studied at $2\times 10^{-11}mol/L$ concentration of Cd^2+^. It can be seen that the transmission spectrum has barely changed within 5 min in Figure 6. This indeed proves that the THF with membrane has a fast response and good time stability.

### Real sample test

Due to the heterogeneity and complicacy of actual samples, the sensing performance of Cd^2+^ in aqueous solution prepared in laboratory might be different from that of the environmental water samples. Thus, it is necessary to investigate the characteristics of the sensor in real environmental samples. Three types of water samples were applied for testing sample’s practical feasibility, including water from two lakes in the university and rainwater. The Cd^2+^ content measurements of the collected real samples were performed according to the above optimal conditions. The calculated Cd^2+^ contents for the noted real samples are displayed in Table 1. The results are basically above 10^−11^ mol/L and RSD lower than 0.47 for five experiments. Compared with the error bar in Figure 5, the RSD corresponding to the actual sample is slightly higher. Considering the influence of other impurities in the water on the binding of the membrane to Cd^2+^, it is normal that the corresponding results are high.

**Table 1:** Test results of Cd^2+^ gradient contents of different real samples

Real sample	Concentration (10ˆ-11 mol/L)	RSD (n = 5)
Lake 1	10	0.47
Lake 2	7	0.21
Rain	9	0.32

### Conclusion

In summary, we have proposed a twisted fiber-based sensing mechanism that can realize high-accuracy *in vitro* detection of Cd^2+^ concentration in water solution. We first demonstrate that the sensing mechanism can be well established on the interference spectrum of coupled core mode and cladding mode. After that, we show the general steps about the fabrication of main sample structure. Experimental measurements prove that the sensitivity of prepared THF with membrane can reach to 0.07252 nm/($10^{-11}mol/L$) in wavelength modulation and 0.08975 dB/($10^{-11}mol/L$) in amplitude modulation. Besides, with bimodal calibration, dual wavelength modulation can be realized to further improve the accuracy of Cd^2+^ concentration monitoring. Finally, no obvious variation of transmission spectrum within 5 min also indicates the good stability of the proposed structure. Without doubt, we believe that such easy preparation, low-cost while high-accuracy and stable as well as environmental-friendly sensing mechanism is promising to be applied in fields such as biochemical devices of Cd^2+^ monitoring, integrated optical sensors, and other related sensing measurement fields.

### Limitations of the study

During the whole experiment, the twisting period of the optical fiber is set by the twisting program in the fusion splicer, so there will be some deviations during the twisting. In addition, while the Cd^2+^ monitoring in the laboratory is very accurate, in actual environment, if the water pollution is too serious, the soil and other particulate impurities in the water have a negative impact on the ions. Although some ions have little effect on the sensor, there will be a blocking effect on the absorption. Finally, in practical application of the sensor, it is still necessary to consider the influence of the external physical conditions and the upper limit of repeatability.

## STAR★Methods

### Key resources table

**Table:** 

REAGENT or RESOURCE	SOURCE	IDENTIFIER
**Chemicals, Peptides, and Recombinant Proteins**		

Acrylamide	Shanghai Macklin Biochemical Co., Ltd	CAS# 79-06-1
1-allyl-2-thiourea	Shanghai Macklin Biochemical Co., Ltd	CAS# 109-57-9
N,N'-ethylenebisacrylamide	Shanghai Macklin Biochemical Co., Ltd	CAS# 110-26-9
2,2-diethoxyacetophenone	Shanghai Macklin Biochemical Co., Ltd	CAS# 6175-45-7
Cd^2+^ solution	Suzhou Reaction Chain New Material Technology Co., Ltd	CAS# 7440-43-9

**Other**		

Single-mode Fiber	Corning Inc	G652D

**Software and Algorithms**		

COMSOL Multiphysics 5.4	COMSOL Inc	https://comsol.com/

### Resource availability

#### Lead contact

Further information and requests for resources and reagents should be directed to and will be fulfilled by the lead contact, Boyao Li (liby@dgut.edu.cn).

#### Materials availability

This study did not generate new unique reagents

### Experimental model and subject details

This study does not use experimental methods typical in the life sciences.

### Method details

#### Twisted fiber preparation

The twisted fiber part was obtained by single-mode fiber (Corning, G652D) operated at special function mode in the fusion splicer (Fujikura, LZM 100) through controlling the z-axis travel length and angle rotation speed of motor at 5 mm and 10 rounds in 90,000 ms.

#### Sensor membrane preparation

Immerse the twisted optical fiber in the sensing membrane solution and slowly rotate the optical fiber for 5 min, then take it out and place it in a drying oven (Shanghai Donglu Instrument and Equipment Co., Ltd., DHG-9030A desktop drying oven). There is a UV curing lamp and a self-made item rotating device inside, and the items are placed on the rotating device. Turn on the UV lamp, set the drying temperature to 45°, and set the drying time to 30 min. Finally, a chiral single-mode optical fiber coated with a sensing film is obtained.

#### Sensor characterization

The Laser signal was injected into the sensor through the light source (wavelength range: 1400nm–1600 nm) of the spectrometer (YOKOGAWA, AQ6370D), and passed through a self-made liquid test chamber (as shown in Figure 3C in the text) and a single-mode pigtail. The final signal is collected by the spectrometer.

#### Gradient Cd^2+^ sample solution preparation

The sample solution is prepared through sample dilution from the standard solution (Suzhou Reaction Chain New Material Technology Co., Ltd., CAS7440-43-9Cd, product specification ICP5 mg/L 100 mL).

#### Numerical simulation

The light field of the twisted fiber was numerically simulated using 'Mode Analysis' in the 'Wave Optics' module in the COMSOL software. Due to the use of torsional coordinate transformation, the model uses 2D end face analysis instead of 3D analysis. The boundary adopts the perfectly matched layer, and the completed mesh division consists of 452020 domain elements and 6410 boundary elements. Limit loss can be viewed in 'ewfd.dampzdB' in option 'surface'. The mode distribution can be viewed in ewfd.normE in option 'surface'.

### Quantification and statistical analysis

This study does not include quantification and statistical analysis.

## Data Availability

Data reported in this paper will be shared by the lead contact upon request. There is no dataset or code associated with this work.
