# Supplementary material for: Twist-assisted high sensitivity chiral fiber sensor for Cd2+ concentration detection
Source: iScience. 2022 Sep 29;25(10):105245. doi: 10.1016/j.isci.2022.105245 (PMC9574500; doi:10.1016/j.isci.2022.105245)
Supplement: Document S1. Figure S1 [file mmc1.pdf]

iScience, Volume *25*

Supplemental information

Twist-assisted high sensitivity chiral fiber sensor for Cd^{2+} concentration detection

Boyao Li, Yaoyao Liang, Aoyan Zhang, Lu Peng, Jinghua Sun, and Guiyao Zhou

Supplementary information

Twist-assisted high sensitivity chiral fiber sensor for Cd^{2+} concentration detection

Boyao Li, Yaoyao liang, Aoyan Zhang, Lu Peng, Jinghua Sun, Guiyao Zhou

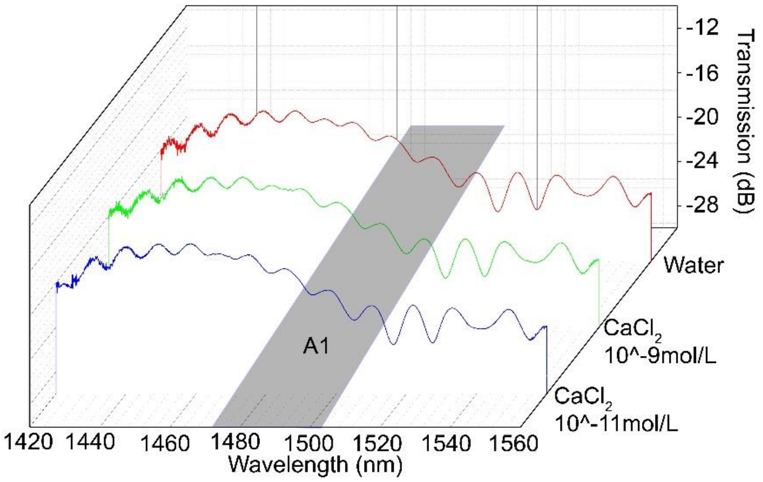

Fig. S1 Transmission characteristics of the sensor in different $CaCl_2$ concentration solutions. Related to **Figure 3**.
